# Aflatoxin Control in Maize by *Trametes versicolor*

**DOI:** 10.3390/toxins6123426

**Published:** 2014-12-17

**Authors:** Marzia Scarpari, Cristiano Bello, Chiara Pietricola, Marco Zaccaria, Luigi Bertocchi, Alessandra Angelucci, Maria Rosaria Ricciardi, Valeria Scala, Alessia Parroni, Anna A. Fabbri, Massimo Reverberi, Slaven Zjalic, Corrado Fanelli

**Affiliations:** 1 Environmental Biology Department, Università Sapienza, Roma 00185, Italy; E-Mails: marzia.scarpari@uniroma1.it (M.S.); chiara.pietricola@live.it (C.P.); marco.zaccaria@uniroma1.it (M.Z.); valeria.scala@uniroma1.it (V.S.); alessia.parroni@uniroma1.it (A.P.); adele.fabbri@uniroma1.it (A.A.F.); corrado.fanelli@uniroma1.it (C.F.); 2 Zooprohylactic Experimental Institute of Lombardia and Emilia Romagna “Bruno Ubertini”, Brescia 25124, Italy; E-Mails: cristiano.bello@tin.it (C.B.); luigi.bertocchi@izsler.it (L.B.); alessandra.angelucci@izsler.it (A.A.); 3 Cellular Biotechnology and Hematology Department, Università Sapienza, Roma 00185, Italy; E-Mail: mariarosaria.ricciardi@uniroma1.it; 4 Department of Ecology, Agronomy and Aquaculture, University of Zadar, 23 000 Zadar, Croatia; E-Mail: slazjl@libero.it

**Keywords:** *Trametes versicolor*, aflatoxins, laccase, cow feed

## Abstract

*Aspergillus flavus* is a well-known ubiquitous fungus able to contaminate both in pre- and postharvest period different feed and food commodities. During their growth, these fungi can synthesise aflatoxins, secondary metabolites highly hazardous for animal and human health. The requirement of products with low impact on the environment and on human health, able to control aflatoxin production, has increased. In this work the effect of the basidiomycete *Trametes versicolor* on the aflatoxin production by *A. flavus* both *in vitro* and in maize, was investigated. The goal was to propose an environmental loyal tool for a significant control of aflatoxin production, in order to obtain feedstuffs and feed with a high standard of quality and safety to enhance the wellbeing of dairy cows. The presence of *T. versicolor*, grown on sugar beet pulp, inhibited the production of aflatoxin B1 in maize by *A. flavus*. Furthermore, treatment of contaminated maize with culture filtrates of *T. versicolor* containing ligninolytic enzymes, showed a significant reduction of the content of aflatoxin B1.

## 1. Introduction

Mycotoxins represent a heterogeneous group of low molecular weight chemical compounds, which present a biological activity, produced in the secondary metabolism by some fungal species, which mainly belong to *Aspergillus*, *Penicillium* and *Fusarium* genera. These secondary metabolites have a cytotoxic, mutagenic and carcinogenic activity in both man and animals and, usually, they can be found as common contaminants into feed and food chains. The development of toxigenic fungi and subsequently the synthesis of mycotoxins may occur in each of the step of these chains, starting from field cultivation up to consumption, passing through storage and preservation. Some mycotoxins can be carcinogenic (fumonisins, FB, Group 2B: possible carcinogen for humans; IARC), carcinogenic and teratogenic (ochratoxin A, OTA, Group 2B, IARC), carcinogenic, mutagenic and teratogenic (aflatoxin B1, AF, Group 1; aflatoxin M1, Group 2B; IARC), [1]. Mycotoxins contamination in cereals intended for human and animal consumption, is a serious food safety issue regarding productions from all over the world. In particular, maize and by-products could be contaminated by different class of mycotoxins including one of the most dangerous to human health and animal found in nature, aflatoxin B1 (IARC) [1].

The major part of the methods for controlling mycotoxin contamination into feed- and foodstuffs has been performed by using chemicals that are hazardous for humans and the environment. The growing interest and awareness toward environmental pollution have addressed the research on the possibility to adopt “green” approaches in the control of fungal contamination and to prevent or detoxify mycotoxins. Due to high economic losses and health hazard issues as consequence of AF contamination, several strategies have been studied and applied to reduce the risk in maize [2]. These strategies can be divided into: (1) stopping the infection process (host plant resistance, biocontrol); (2) pre-harvest crop management practices (good agricultural practices) and (3) post-harvest management strategies (timely-harvesting, properly drying). The former consists in finding the source of resistance in maize germplasm with natural resistance to *A. flavus* infection (pre-harvest resistance). As a result, many new sources of resistance were identified or released, such as Mp420, Mp313E, Mp715, Mp717 and GT-MAS:gk, CI2, MI82, and Tex6. These lines tend to possess undesirable agronomic characteristics [3], and cannot be used directly to breed for commercial aflatoxin resistant maize varieties. Recently, -omic approaches boosted the findings of novel gene target for selecting maize resistance to *A. flavus* and/or aflatoxins [4,5] even if a concrete result has not been yet achieved. Breeding programs exist to develop new varieties and to replace varieties that have lost their resistance, but the maintenance cost of this system is high. Agricultural scientists recognized the potential negative consequences of planting large areas to single, uniform crop cultivars as early as the 1930s. Thus, an Integrated Pest Management (IPM) approach is a widely recognized ecosystem approach to crop production and protection that combines different management strategies and practices to grow healthy crops and minimize the use of pesticides with considerable success. The same concept could be easily transferred to aflatoxin management, in which complementary, low cost and efficient strategies can be combined to control toxin contamination in maize. In relation to this, several sustainable strategies have been/are currently established for answering to this demand. Notably, biocontrol agents and predictive models have been recently developed for controlling aflatoxin in the pre-harvest [6,7].

Disease management programs to risk use predictive modeling tools-stratify members in order to optimize the utilization of available pest management resources. Focusing the research on a time scale of one decade—by taking into account environmental and monitoring data from past research—may lead to create and apply predictive models that can forecast the spread of main pests and diseases of maize. These models integrate natural and social systems because examine a variety of coupled interactions (insects, maize and fungal pathogens) and feedbacks among relevant systems (food safety and production yields) [8]. These types of mechanistic models have been successfully applied to prevent *A. flavus* contamination in maize [6].

Over recent years, biocontrol agents able to challenge specific pathogens in maize have been selected [7,9,10]. Some of them pose cross-contamination risk (*i.e*., introducing a microorganism safe in a determinate environment but unsafe for another) and some other are not stable in the wild (sexual recombination amongst vegetative incompatible selected strains), [11]. Alternatively, and more safely, bioactive compounds from biocontrol agents may be used for enhancing plants defences and/or for inhibiting pathogen growth and/or toxin synthesis [12].

In this paper, we propose two valid, low cost methods for safely containing aflatoxin contamination into maize kernels at large scale. The former concerns the optimization and large-scale production of an exo-polysaccharides mixture originated by *Trametes versicolor* while the second regards the attempt to use detoxifying agents, namely lignin degrading enzymes from *T. versicolor*, in feed composition for abating AFs content.

## 2. Results and Discussion

### 2.1. Inhibition of Aflatoxin B1 in Liquid Culture of A. flavus by LF and LS of T. versicolor

In previous works, we demonstrated that bioactive compounds secreted in the culture filtrates of the white rot basidiomycete *T. versicolor*, were able to stimulate the antioxidant system of pathogenic fungi, thereby inhibiting the production of mycotoxins [13,14]. In this study, we approach the problem from a different point of view. In fact, previous studies were conducted using expensive media for growing fungi and obtaining bioactive compounds, which were herein substituted with plant wastes.

**Figure 1:**
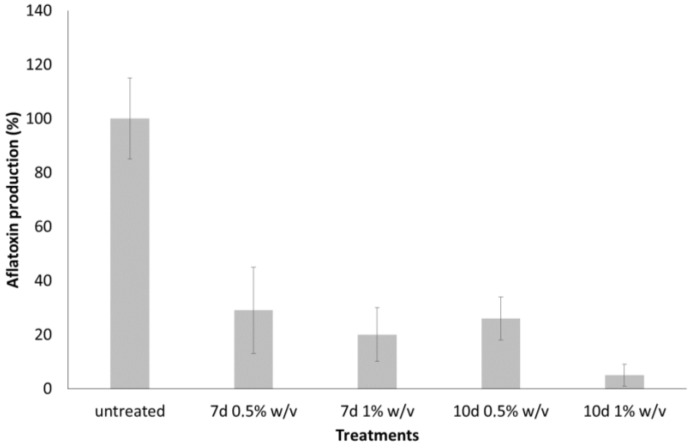
Effect of different concentration (0.5%, 1% *w*/*v*) of lyophilized filtrates (LF) from *T. versicolor* CF117 cultured on molasses for 7 and 10 days on aflatoxin B1 production by *A. flavus* (“*in vitro*” experiments) after 5 days of incubation at 30 °C. The data are the mean ± SD of three determinations of three separate experiments.

The data collected from the study performed on the strain used in this work, confirm the ability of *T. versicolor* CF117 culture filtrate, to inhibit the production of aflatoxin by *A. flavus* in liquid cultures and in maize seeds. An inhibition of aflatoxin B1 production, ranging from 70% to 90% (*p* < 0.001), in *A. flavus* treated with LF from *T. versicolor* CF117 strain occurred (Figure 1). Aflatoxin B1 biosynthesis inhibition, pinpointed by liquid filtrate (LF) of *T. versicolor* grown on molasses (Figure 1), was comparable with that obtained with LF from Potato Dextrose Broth (PDB) culture reported [14].

**Figure 2:**
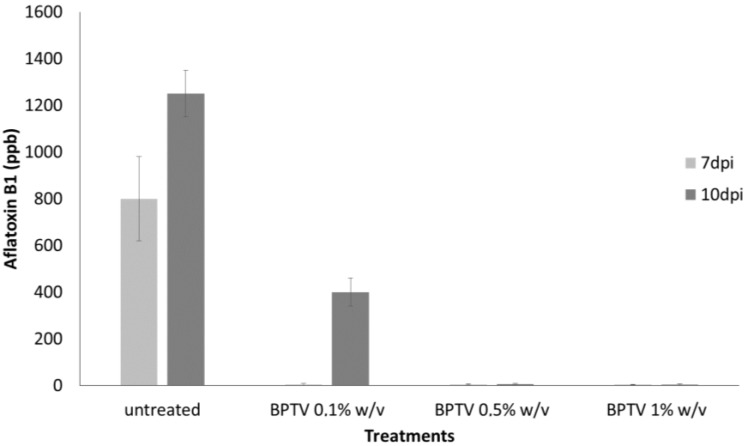
Effect of different concentration (0.1%, 0.5%, 1% *w*/*v*) of lyophilized solid from *T. versicolor* CF117 grown in sugar beet pulp substrate (BPTV) on aflatoxin B1 production by *A. flavus* (“*in vitro*” experiments) after 7 and 10 days post inoculation (dpi) at 30 °C. The data are the mean ± SD of three determinations of three separate experiments.

The ability of *T. versicolor*, grown on solid substrate, to inhibit the production of aflatoxin B1, was also investigated. Lyophilized solid (LS) of *T. versicolor* CF117 inoculated on beet pulp (BPTV), was added to a liquid culture of *A. flavus* at 0.1, 0.5 and 1% *w*/*v*. The results showed (Figure 2) that the LS added at 1% *w*/*v* was able to inhibit completely (*p* < 0.01) the production of aflatoxin B1 up to 7 days post inoculation (dpi) and to reduce dramatically fungal growth (data not shown). Results are significant (*p* < 0.01) up to 10 dpi also by adding minor amounts of LS, *i.e.*, LS added at 0.1% and 0.5% *w*/*v*, showed a significant percentage of aflatoxin inhibition.

### 2.2. Inhibition of Aflatoxin B1 in Maize Kernels Contaminated by A. flavus by LF and LS of T. versicolor

**Figure 3:**
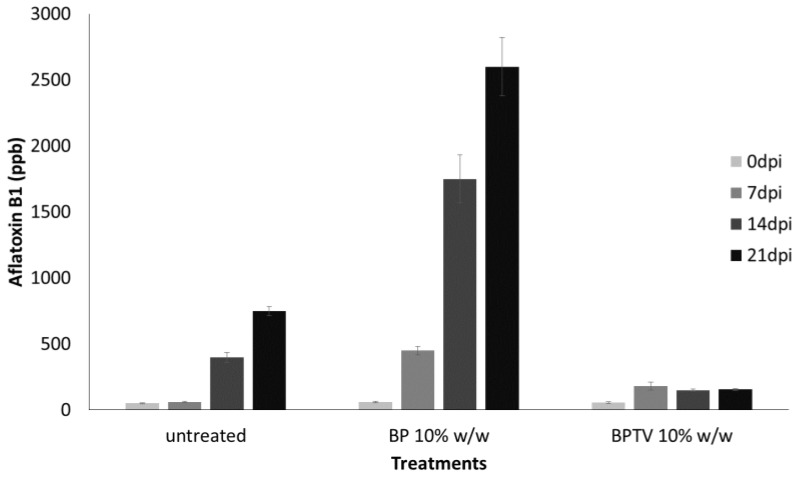
Effect of lyophilized solid from *T. versicolor* CF117 grown in sugar beet pulp substrate (BPTV; 10% *w*/*w*) or the sole sugar beet pulp substrate (BP; 10% *w*/*w*) on aflatoxin B1 production by *A. flavus* (“*in vivo*” experiments) after 0, 7, 14, 21 days post inoculation (dpi) in maize kernels at 30 °C. The data are the mean ± SD of three determinations of three separate experiments.

Figure 3 showed that the treatment with BPTV from *T. versicolor* CF117 strain inhibited aflatoxin B1 formation in maize kernels inoculated with *A. flavus* (Figure 3) up to 21 dpi (*p* < 0.001). Significant inhibiting effects of BPTV on maize make the application of these products attractive to control aflatoxins in seeds.

Moreover, the addition of the assayed LF and LS could contribute to enhance the nutritional value of feed; in fact, they contain nutritive and active compounds. Some of these substances, such as β-glucans, are able to affect beneficially different target functions in animals in such way that the state of well-being, health and resistance to disease are improved [15]. The marked inhibition of aflatoxin biosynthesis, demonstrated by LF and LS on both liquid culture and maize seeds, allowed considering its application for possible use on a large scale. Furthermore, production of LF and BPTV from *T. versicolor* has been achieved using molasses and sugar beet pulps wastes from sugar beet processing. The choice of a growth substrate composed of molasses (liquid culture) or sugar beet pulps (solid culture), is also due to the possibility to add LF or BPTV straight in the composition of the livestock feed, in order to protect it from aflatoxins contamination by *A. flavus* during storage and in the mangers.

### 2.3. Degradation of AFB1 by Lignin Degrading Enzymes of T. versicolor

*T. versicolor,* as well as other white rot fungi, produces also laccases [16,17,18], enzymes capable of degrading lignin. The fungal laccase enzymes are very interesting as they are “green catalysts” using oxygen as oxidant and produce water as a reaction by-product [19]. Laccases have a low substrate specificity that allows them to oxidize a large number of organic and inorganic compounds. Moreover, laccases have also the ability to degrade aflatoxin B1 by a still unknown mechanism [20]. It was therefore studied the ability of culture filtrates of *T. versicolor*—enriched in laccases, to degrade aflatoxin B1 present in contaminated matrices, hence providing an efficient and secure method of maize detoxification.

**Figure 4:**
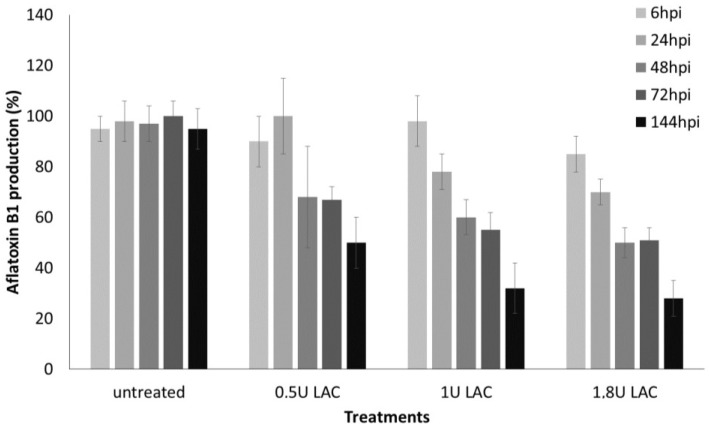
Effect of culture filtrate displaying different laccase activity (0.5, 1.0, 1.8 U/mL) from *T. versicolor* CF294 on aflatoxin B1 degradation after 6, 24, 48, 72, 144 h post inoculation (hpi) at 30 °C. The data are the mean ± SD of three determinations of three separate experiments.

In previous unpublished studies, *T. versicolor* CF294 strain has been identified as the best laccase producer among strains collected worldwide. Figure 4, pinpointed the ability of CF294 culture filtrate, displaying a laccase activity of 1.8 U/mL, to degrade—under *in vitro* conditions, up to 70% of aflatoxin B1 after 48 h (*p* < 0.01).

Under *in vivo* conditions, the culture filtrate was sprayed over previously contaminated maize seeds without altering significantly their moisture level. The nebulization method allowed a homogeneous distribution of the product in the kernels bulk. In Table 1, it was reported the degradation values obtained by treating 0.1 kg of *A. flavus-*inoculated maize kernels, with the culture filtrate of CF294 having laccase activity of 3.5 U/mL. The best performance “70% of toxin degradation” was achieved using the culture filtrate added at 2% *v*/*w* (*p* < 0.01). At larger scale, 30Kg of naturally contaminated maize seeds and milled maize seeds were treated with culture filtrate of CF294, having laccase activity 3.5 U/mL. Table 2 showed the values of the percentage of degradation of aflatoxin B1, after treatment with culture filtrate. Both treated samples (seeds and milled seeds) showed a significant degradation of aflatoxin B1 content after 7 days of the treatment up to 30% for seeds and 43% for milled seeds (*p* < 0.01 and *p* < 0.05 respectively). These data obtained *in vitro* (liquid) and *in vivo* (maize) experiments indicate that culture filtrates of CF294 was able to reduce significantly aflatoxin content.

**Table 1:** Effect of culture filtrate of *T versicolor* CF 294 containing 3.5 U or 7 U of laccase (LAC) on the degradation of aflatoxin B1 in maize samples previously inoculated with *A. flavus*. Aflatoxin B1 was determined after 96 h by HPLC-DAD. Data are the mean ± SD of six replicas for each treatment condition.

Sample	Aflatoxin B1 (ppb)	% Degradation
untreated	1528 ± 250	-
3.5 U LAC	760 ± 52	50 ± 6
7 U LAC	458 ± 40	70 ± 11

**Table 2:** Effect of culture filtrate of *T. versicolor* CF 294 containing 1050 U of laccase on the degradation of aflatoxin B1 in maize samples naturally contaminated with aflatoxin B1. Determination of aflatoxin B1 was performed after 7 and 14 days by HPLC-DAD. Data are the mean ± SD of 6 replicas for each treatment condition.

Time (days)	Aflatoxin B1 (ppb)			
	Seeds	% degradation	milled seeds	% degradation
0	74 ± 5	-	140 ± 4	-
7	53 ± 3	30 ± 6	80 ± 2	44 ± 3

### 2.4. Human Cell Viability Assays

**Figure 5:**
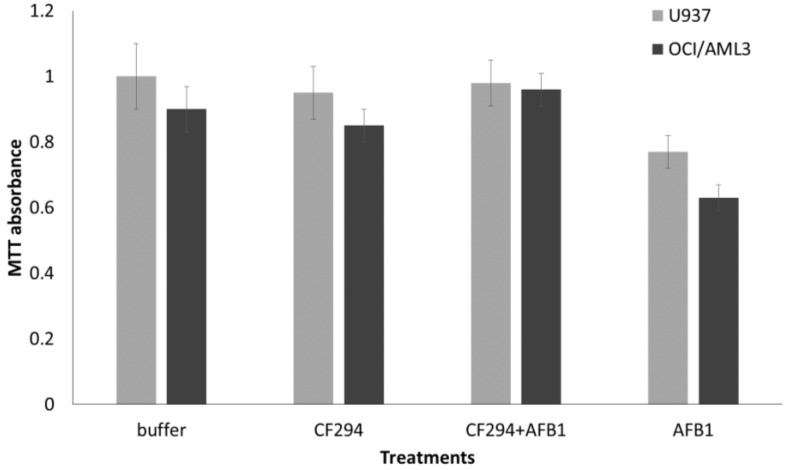
MTT assay for assessing cell viability. Human lymphoma (U937) and myeloid leukemia cells were cultured up to 48 hours with BES buffer (buffer), culture filtrate of CF294 displaying a laccase activity of 3.5 U/mL (CF294), 150 ng of AFB1 incubated for 72 h with the culture filtrate of CF294 displaying a laccase activity of 3.5 U/mL (CF294 + AFB1) or with the sole AFB1 (150 ng). All the experiments were performed at least three times and results were expressed as mean ± standard deviation (SD).

The by-products of AFB1 degradation by laccase have been tested for their putative toxicity on two human lymphoma cancer cells such as OCI-AML3 and U937, being AFB1 known for their cytotoxicity against human immune system cells. The complete degradation of 150ng of AFB1 was performed using the culture filtrate of TV294 having laccase activity of 3.5 U/mL for 72 h at 25 °C. The complete degradation of AFB1 was analysed by HPLC as described above (data not shown). The viability of human cell lines was tested by MTT assay. Results indicated that 150 ng of AFB1 presented a slight, but significant (*p* < 0.01) toxicity on OCI-AML3 and U937 cells compared to the control (buffer) (Figure 5). AFB1 by-products originated from laccase treatment did not affect cell viability compared to the control and to the sole AFB1 (*p* < 0.05).

Concluding, *T. versicolor* strains may represent useful tools for the integrated control of aflatoxins in food and feed maize-derived. The use of culture filtrates of the basidiomycete *T. versicolor* for inhibiting the production and/or to degrade aflatoxin B1 in maize could be a powerful and environmentally friendly tool to reduce the contamination of food commodities by this carcinogenic compound. This would allow obtaining feed with high standards of quality and safety by improving the health and well-being of the animals. Moreover, the use of molasses and sugar beet pulp as growth substrate makes this biocontrol strategy cost-effective and applicable at large scale.

## 3. Materials and Methods

### 3.1. Fungal Strains

*Trametes versicolor* strains CF 117, CF 294 were obtained from the collection of the Laboratory of Plant Pathology, Department of Environmental Biology, University “La Sapienza” of Rome. The strains were cultured on Potato Dextrose Agar (PDA, Biolife, Milan, Italy) in Petri dishes incubated at 25 °C for 7 days. Ten-day liquid cultures of Potato Dextrose Broth (PDB, Himedia, Mumbai, India) of different isolates were prepared from Petri dishes and used as inoculum.

*Aspergillus flavus* (NRRL 3357), producer of aflatoxin B1, was provided by the laboratory of Payne, G.A. of North Carolina State University (Raleigh, NC, USA). The isolate was cultured on PDA at 30 °C for 7 days and a suspension of 1 × 10^6^ conidia per mL of sterilized distilled water was used as inoculum.

### 3.2. Assays of T. versicolor Culture Filtrates on Aflatoxin Production: “In vitro Experiments”

A solution of molasses (from sugar beet processing, kindly provided by COPROB, Minerbio, Italy) and yeast extract (Biolife, Milan, Italy) in a concentration of 30 g/L and 2 g/L respectively, was inoculated with 10% *v*/*v* of homogenized mycelia of *T. versicolor* CF117 isolate. The fungal cultures were incubated at 25 °C in rotary shaken conditions (150 rpm) for 7 and 10 days. The mycelium was separated from the culture medium by filtration through 0.45 µm filters (Sartorius, Goettingen, Germany) and the filtrate was lyophilized (lyophilized filtrate, LF). The LF was added (final concentration 0.5% and 1% *w*/*v*) to 1mL of PDB in 10 mL tubes flasks. The tubes were inoculated with *A. flavus* conidia, as reported above and incubated at 30 °C for 5 days. After incubation, aflatoxin B1 were monitored in the culture filtrates by Agilent 1200 HPLC-DAD (Agilent, Waldbronn, Germany).

### 3.3. Assays of T. versicolor Solid Substrate on Aflatoxin Production: “In vitro Experiments”

Ground beet pulp (from sugar beet processing, kindly provided by COPROB, Minerbio, Italy) was used as non-inert solid support for growing *T. versicolor*. One Kg of sugar beet pulp (BP) was autoclaved at 120 °C for 20 min in a plastic bag with filter strips (Mycelia BVBA, Nevele, Belgium) and inoculated with 1 L of *T. versicolor* CF117 suspension, grown in molasses solution (see above) for 10 days. The bags were incubated at 25 °C for 10 days and the solid substrate was lyophilized (lyophilized solid, BPTV). BPTV was added (final concentration 0.1%, 0.5% and 1% *w*/*v*) to 1 mL of PDB in 10 mL tubes flasks. The tubes were inoculated with *A. flavus* conidia, as reported above and incubated at 30 °C for 7 and 10 days. At 7 and 10 days after inoculation (dpi), aflatoxin B1 were monitored in the culture filtrates by HPLC-DAD (Agilent, Santa Clara, CA, USA) as previously described [21] with modifications (described below).

### 3.4. Assays of T. versicolor Culture Filtrates on Aflatoxin Degradation: “In vitro Experiments”

Thirty-five mL of the following culture medium, glucose, 5 g/L; asparagine, 1 g/L; K_2_HPO_4_, 1 g/L; yeast extract, 0.5 g/L; MgSO_4_℘7H_2_O, 0.5 g/L; KCl, 0.5 g/L; FeSO_4_, 1 g/L; MnSO_4_, 0.008 g/L; ZnSO_4_, 0.003 g/L; CuSO_4_·5H_2_O, 0.03 g/L; NH_4_NO_3_, 1 g/L was inoculated with *T. versicolor* CF294 5% *v*/*v* and incubated at 25 °C. After 3 days, the entire biomass was transferred into 3 L of the same culture medium without glucose, with the addition of 2,5-xylidine 50 µM. The addition of xylidine and the absence of glucose in the growth substrate, create conditions that are conductive for the production of laccase [22]. The culture were incubated for further 7 days at 25 °C. The mycelium was separated from the culture medium by filtration through 0.45 µm filters and the filtrate was stored at 4 °C. 300 ng of aflatoxin B1 (Sigma, Milan, Italy) was added to 500 µL of culture filtrate with different laccase activity (adjusted by diluting culture filtrate with distilled water) and incubated for 6, 24, 48, 72, 144 h. Control was represented by aflatoxin B1 dissolved in PBD solution. Aflatoxin B1 content wad determined by HPLC-DAD (Agilent, Santa Clara, CA, USA) as previously described [21] with modifications (described below).

### 3.5. Experiments on Maize and Seeds

Preparation of a stock of contaminated maize with *A. flavus*: 100g of sterilized maize seeds were inoculated with 1 × 10^6^ conidia of *A. flavus*. The samples were incubated at 30 °C for 7 days. Three grams of *A. flavus* inoculated maize were added to 27 g of sterilized maize and treated with 3 g of BPTV (see above). The samples were incubated at 30 °C for 7, 14 and 21 days. Aflatoxin B1 content was determined by HPLC-DAD as previously described [21] with modifications (described below).

### 3.6. Assays of T. versicolor Culture Filtrates on Aflatoxin Degradation: “In vivo Experiments”

At lab scale, 100 g of maize seeds (see above) were placed on plastic sheets and were treated, *via* nebulizer, with 1 or 2 mL of *T. versicolor* CF294 culture filtrate having laccase activity of 3.5 U/mL. The plastic sheets were then closed, leaving an opening for the exchange of air with the outside. The sheets were incubated at 30 °C for 96 h. At large scale, 30 Kg of maize seeds and 30 Kg of milled maize seed, were treated with *T. versicolor* CF294 culture filtrate having laccase activity of 3.5 U/mL. A nebulizer both on the seeds and on the flour provided 300 mL of culture filtrate per 30 Kg of seeds. The samples were kept at room temperature for 7 and 14 days. Aflatoxin B1 content was determined by HPLC-DAD as previously described [21] with modifications (described below).

### 3.7. Cell Viability Assays

For testing if the AFB1 fragments originated following 72 h-incubation at 25 °C with 3.5 U/mL of CF294 culture filtrate having laccase activity affect cell viability, human OCI-AML3 and U937 (0.3 × 10^6^/mL) cells were seeded into 24-well plates and incubated for 48 h with the different samples following which 100 µL of each condition were transferred in triplicate to a 96-well plate (500 μL/well). The cells were incubated in presence of 50 μL of buffer BES (25 mM BES; 250 mM NaCl; pH 6.0), or of buffer containing CF294 culture filtrate having laccase activity of 3.5 U/mL, or of buffer containing CF294 culture filtrate having laccase activity of 3.5 U/mL which have reacted for 72 h with 150 ng of AFB1 at 25 °C, or of buffer containing 150 ng of AFB1. Then, 10 µL of MTT [3-4,5-dimethylthythiazol-2-yl)-2,5-diphenyltetrazolium bromide] (Sigma-Aldrich, Milan, Italy) were added to each well and plates were incubated for 3 h at 37 °C with 5% CO_2_. After 3 h, MTT was dissolved with 100 µL of solubilization solution and absorbance was measured at 570 nM with a spectrophotometer. All the experiments were performed at least three times and results were expressed as mean ± standard deviation (SD).

### 3.8. Analytical Determination

One hundred milliliters of a solution of methanol-water 80:20 *v*/*v*, were added to 50 g of maize seeds and 5 g of NaCl. The solution was blended at high speed for 1 min and 10 mL of the extract were diluted with 40 mL of water. Ten milliliters of the diluted solution were filtered through 0.45 μm filter and purified by immunoaffinity column (Aflatest, Vicam, Boston, MA, USA). Aflatoxin B1 was eluted with 1mL of methanol (Sigma, Milan, Italy), dried and the residue was dissolved with 200 μL of methanol. Aflatoxin B1 content was determined by HPLC-DAD equipped with a reversed phase Zorbax-Aq C18 column (150 × 2.0 mm I.D, 3.5 μm) thermostated at 30 °C. The separations were performed by gradient elution of increasing concentration of acetonitrile (Romil, Cambridge, UK) in water (Romil, Cambridge, UK), both acidified with 1% *v*/*v* of formic acid, at a flow rate of 0.2 mL/min. Detection was performed at 363 nm. For the quantification of aflatoxin B1 in different matrices, a calibration curve was constructed using standard aflatoxin B1 at different concentrations. The identification and quantification of aflatoxin B1 were performed respectively based on its retention times and spectroscopic spectrum and by the external standard method using a six point regression graph of the UV-visible absorption data collected at 363 nm.

### 3.9. Laccase Activity

The enzymatic activity of laccase was assayed by the method described by Harkin and Obst [23]. To 2.5 mL of a solution of 50 mM sodium acetate at pH 4.5, were added 0.4 mL of a 0.5 M solution of syringaldazine (Sigma, Milan, Italy) in ethanol; 100 µL *T. versicolor* CF 294 culture filtrate were added to the solution and the reaction is monitored by UV-vis spectrophotometer (Beckman DU530 UV/VIS). Oxidation of syringaldazine was monitored through absorbance increase at 525 nm (ε = 65,000 M^−1^ cm^−1^). One unit of enzyme activity was defined as the amount of enzyme required to oxidize 1 μM of syringaldazine per min^−1^.

### 3.10. Statistical Analysis

The results obtained in the present investigations were subjected to statistical analysis using SPSS package 12.0 version. We computed statistical parameters mean, S.D, S.E and *t*-value are interpreted at α = 0.05 level of significance.
